# Inconsistent definitions of prolonged labor in international literature: a scoping review

**DOI:** 10.1016/j.xagr.2024.100360

**Published:** 2024-06-05

**Authors:** Wouter Bakker, Evelien M. Sandberg, Sharon Keetels, Jan W. Schoones, Monica Lauridsen Kujabi, Nanna Maaløe, Salome Maswime, Thomas van den Akker

**Affiliations:** 1 Athena Institute, VU University, Amsterdam, The Netherlands; 2 Department of Obstetrics and Gynecology, Leiden University Medical Center, Leiden, The Netherlands; 3 Directorate of Research Policy, Leiden University Medical Center, Leiden, The Netherlands; 4 Global Health Section, Department of Public Health, University of Copenhagen, Denmark; 5 Department of Obstetrics and Gynecology, Aarhus University Hospital – Skejby Hospital, Aarhus, Denmark; 6 Department of Obstetrics and Gynecology, Copenhagen University Hospital – Herlev Hospital, Copenhagen, Denmark; 7 Global Surgery Division, Department of Surgery, University of Cape Town, Cape Town, South Africa

**Keywords:** active support of labor, definition, labor dystocia, labor management, labor progress, partograph, prolonged first stage of labor, prolonged second stage of labor

## Abstract

**Objective:**

Prolonged labor is the commonest indication for intrapartum cesarean section, but definitions are inconsistent and some common definitions were recently found to overestimate the speed of physiological labor. The objective of this review is to establish an overview of synonyms and definitions used in the literature for prolonged labor, separated into first and second stages, and establish types of definitions used.

**Data sources:**

A systematic search was conducted in PubMed, Embase, Web of Science, Cochrane Library, Emcare, and Academic Search Premier.

**Study eligibility criteria:**

All articles in English that (1) attempted to define prolonged labor, (2) included a definition of prolonged labor, or (3) included any synonym for prolonged labor, were included.

**Methods:**

Data on study design, year of publication, country or region of origin, synonyms used, definition of prolonged first and/or second stage, and origin of provided definition (if not primarily established by the study) were collected into a database.

**Results:**

In total, 3402 abstracts and 536 full-text papers were screened, and 232 papers were included. Our search established 53 synonyms for prolonged labor. Forty-three studies defined prolonged labor and 189 studies adopted a definition of prolonged labor. Definitions for prolonged first stage of labor were categorized into: time-based (*n*=14), progress-based (*n*=12), clinician-based (*n*=5), or outcome-based (*n*=4). For the 33 studies defining prolonged second stage, the majority of definitions (*n*=25) were time-based, either based on total duration or duration of no descent of the presenting part.

**Conclusions:**

Despite efforts to arrive at uniform labor curves, there is still little uniformity in definitions of prolonged labor. Consensus on which definition to use is called for, in order to safely and respectfully allow physiological labor progress, ensure timely management, and assess and compare incidence of prolonged labor between settings.

**AJOG Global Reports at a Glance**

**Why was this study conducted?**

Prolonged labor is the commonest indication for intrapartum cesarean section, but definitions are inconsistent.

**What are the key findings?**

In 232 studies, we found 53 synonyms for prolonged labor and a large variety in definitions. Definitions of prolonged first stage of labor are categorized in time-based, progress-based, clinician-based, or outcome-based. The majority of definitions for prolonged second stage of labor are time-based, either based on total duration or duration of no descent of the presenting part.

**What does this study add to what is already known?**

This study summarized and categorized the substantive amount of different definitions of prolonged labor used in the literature, portraying the huge inconsistencies and underlining the need for clarity and uniformity.

## Introduction

Prolonged labor is the commonest indication of intrapartum cesarean section worldwide, but simultaneously one of the most challenging conditions to identify and manage.^1^, ^2^, ^3^, ^4^, ^5^, ^6^ Whilst a lot of studies have been conducted into prolonged labor and its outcomes, evidence on how to prevent and treat prolonged labor is more challenging to navigate and seems to be conflicting in nature. Studies show that in several settings around the world, there is a harmful overuse of synthetic oxytocin and cesarean section to manage prolonged labor, whilst underuse is present in other settings.^7^, ^8^, ^9^

Different perspectives exist on the physiological labor progress. Historically, Friedman's research was crucial in establishing a baseline to define labor progress in nulliparous and multiparous women, and still forms the foundation for many labor management protocols today.^10^, ^11^, ^12^ Based on Friedman's labor curves, Philpott designed a labor monitoring tool we now know as the partograph (also partogram).^13^ In more recent years, the establishment of a Consortium on Safe Labor contributed to a change in perspective on labor progress.^14^, ^15^, ^16^, ^17^ Although this led to updates of several national guidelines for prolonged labor, and the World Health Organization (WHO) adjusting its long-used partograph into a new Labor Care Guide allowing for a longer course of labor, these insights have not yet been adopted universally and have also been met with criticism.^18^, ^19^, ^20^, ^21^, ^22^, ^23^

This suggests a lack of uniform international language to describe prolonged labor, since at present multiple case definitions can be found in guidelines and clinical studies.^24^ Problems to arrive at a uniform definition include the difficulty to determine labor onset and different labor stages, and problems to stratify by parity, maternal medical history or use of regional anesthesia.^25^^,^^26^ Moreover, in the literature, different terms such as prolonged labor, cephalopelvic disproportion (CPD) and obstructed labor are used interchangeably, while CPD is actually better described as a cause, and obstructed labor as an extreme outcome of prolonged labor.^27^^,^^28^

A standardized and practical case definition is required to compare incidence and outcomes, alongside uniform clinical guidance to improve management of prolonged labor. As a first step, to establish an overview of the terminology used in prolonged labor research, we performed a scoping review summarizing the definitions and categorizing these in themes.

## Materials and methods

### Protocol and registration

The protocol for this study was registered in the International Prospective Register of Systematic Reviews (PROSPERO) with registration number CRD42023407040. This review was conducted in accordance with the Preferred Reporting Items for Systematic Reviews and Meta-Analyses (PRISMA) statement.

### Research question

The main research question is: how is prolonged labor defined in the international scientific literature? An overview of all synonyms used in the literature is given, as well as a categorization by theme.

### Search strategy

We undertook a systematic search of the literature to identify papers that defined prolonged labor or a synonym. The full search strategy is provided in Appendix 1. Where applicable, predefined search terms were used. We searched the following databases: PubMed, Embase, Web of Science, Cochrane Library, Emcare, and Academic Search Premier. No filter for publication year was applied. We decided to only include original research studies, such as randomized controlled trials, and retrospective and prospective cohort studies. We decided to exclude (generally secondary-derived) guidelines, literature reviews, commentaries, and study protocols. Studies from all geographic areas and settings were included. Only studies with a full-text report available in English were included. Search results were imported into Clarivate Endnote 20.5.

### Study selection

**Figure 1:**
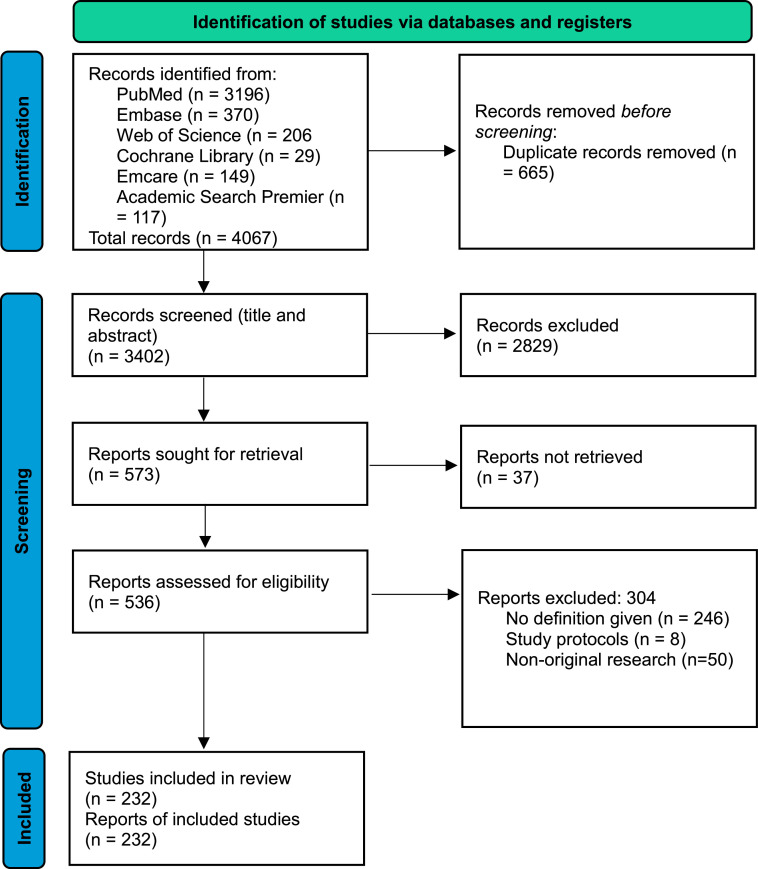
PRISMA flowchart for this study. Adapted from: Page et al. 2021.^30^

After removal of duplications results were screened based on title and abstract by at least two different researchers (WB, ES, and SK). After selection of abstracts, full-text articles were retrieved and read. Each paper was reviewed by at least two out of three researchers (WB, ES, and SK). Disagreements were discussed amongst these three researchers, with TvdA as a consultant. Through reference checking a few additional key publications were identified and added. Moreover, the search strategy was updated and optimized, since new synonyms were identified during the initial searches. The ultimate search was performed on May 30, 2023. Figure 1 shows the PRISMA flowchart of the selection process.

Bakker. Scoping review on definition of prolonged labor. Am J Obstet Gynecol Glob Rep 2024.

### Assessment of risk of bias

A scoping review design was used, which is better suited to create an overview in a vast and diverse body of evidence compared to a systematic review, since we aimed to scope the diverse body of literature for available evidence regarding definitions of prolonged labor.^29^ A systematic search in line with systematic review protocols was performed, but a structured risk of bias analysis was not performed.

### Data synthesis

The following data were extracted from the full-text publications, if available and applicable to the type of study: year of publication, country or region of origin, study design, synonym used, definition of prolonged (active) first and/or second stage, and if not primarily established by the study itself the reference of the used definition. Four items were analyzed as part of the definition of prolonged labor: use of a partograph, evaluation of uterine contractions, use of oxytocin augmentation, and ruptured membranes (spontaneous or artificial). Data were collected in Microsoft Excel 16.74.

### Core outcome sets

The COMET (Core Outcomes Measures in Effectiveness Trials) and CROWN (Core Outcomes in Women's Health) initiatives report no core outcome set for prolonged labor. Therefore, this study did not make use of a core outcome set.

### Patient involvement

No patients were directly involved in the development of this review, but some of the included studies involve women's perspectives on prolonged labor management. The need for this review came from clinicians from different geographical areas struggling with the identification of prolonged labor in the women they care for, to provide the optimal intervention at the right moment.

## Results

### Study selection

A total of 4067 studies were identified, of which 665 were duplicates. The remaining 3402 studies were screened for title and abstract, whereby 2829 studies were excluded. Retrieval was sought for 573 articles: 37 full texts could not be retrieved, despite attempts to contact the authors. In total, 536 articles were assessed for eligibility, of which 246 were excluded as no definition was given in the report. Another 58 were excluded because these were not original research reports.

### Study characteristics

**Figure 2:**
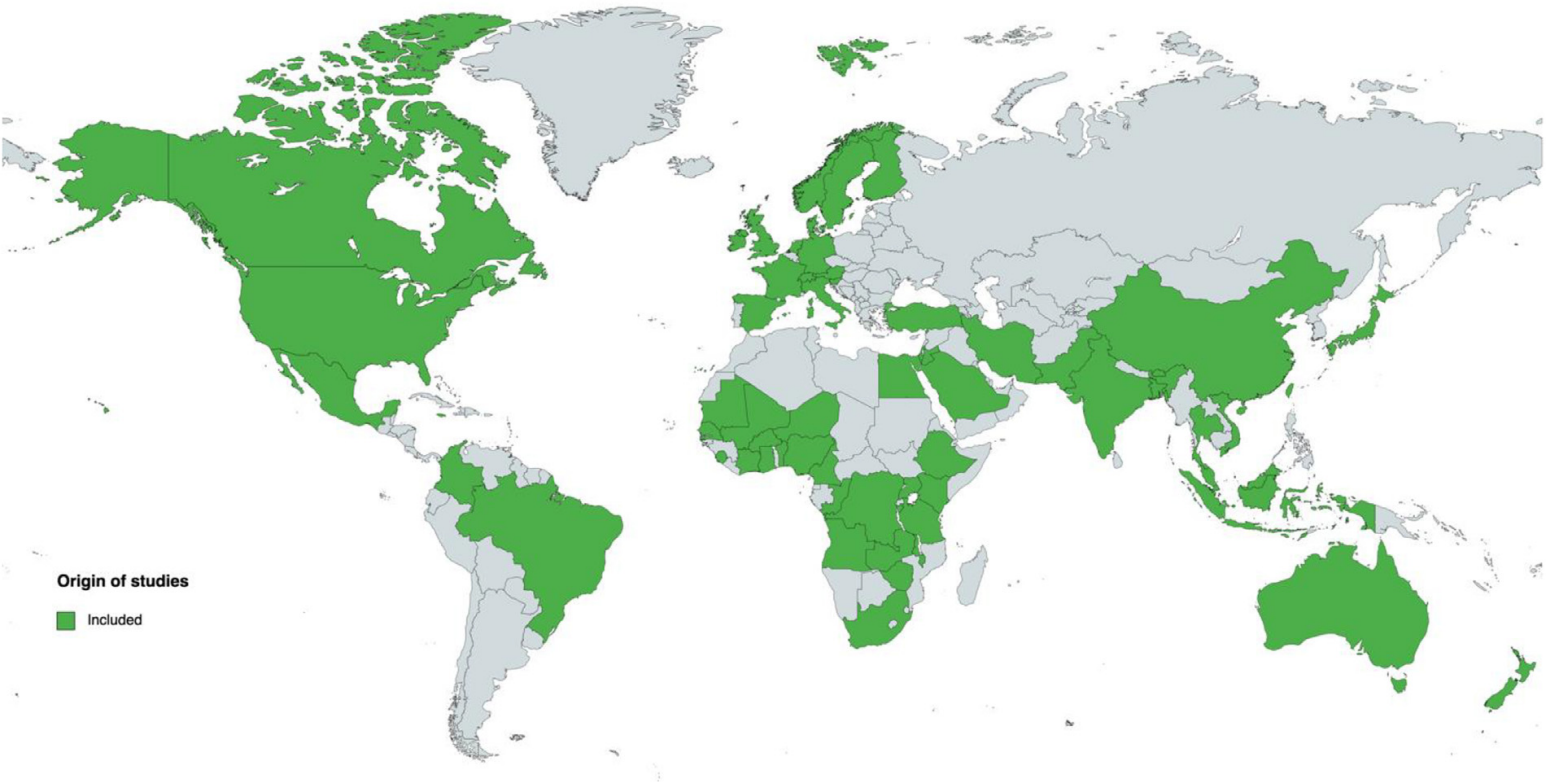
Overview of countries of origin of the included studies. Created with mapchart.com.

We included 232 original research studies in this review, originating from all continents, with a predominance of the USA (*n*=60), Norway (*n*=18), Sweden (*n*=18), United Kingdom (*n*=14), and Israel (*n*=14) (Figure 2). Years of publication ranged from 1980 (*n*=1)^31^ to 2023 (*n*=2),^32^^,^^33^ with 135 (58.1%) studies from the last decade (2014–2023). The majority were observational studies (192, 82.8%), 23 (9.9%) were randomized controlled trials. We then stratified studies into two groups: (1) studies primarily attempting to define prolonged first and/or second stage of labor or any synonym (*n*=43) and (2) those with other primary aims, but adopting predefined criteria for prolonged labor or any synonym in the analysis of outcomes (*n*=189).

Bakker. Scoping review on definition of prolonged labor. Am J Obstet Gynecol Glob Rep 2024.

### 1. Synonyms of prolonged labour

Table 1 shows the 53 synonyms of prolonged labor identified in the included studies. We identified seven major categories that we titled “prolonged labor,” “labor arrest,” “dystocia,” “protracted labor,” “failure to progress,” “delay in labor,” and “abnormal labor.” Terms that did not seem to fit into any of these categories were listed under a category “other,” where CPD and obstructed labor were also placed. Some studies make use of very specific definitions, for example, primary and secondary dystocia or mildly and very protracted labor, or differentiate between synonyms, for example, between protraction and arrest.^34^, ^35^, ^36^ Other studies used such terms interchangeably or adhered to one specific term.^37^^,^^38^

**Table 1:** All terminology related to prolonged labor identified in the included studies, grouped into main denominators

**Prolonged** Prolonged labor Prolonged active phase of labor Prolonged first stage of labor Prolonged second stage of labor (often PSSL) **Arrest** Arrest disorders Arrest of labor Arrested labor Arrest of dilation/dilatation Arrest of first stage Arrest of second stage Arrest of progress Arrest of descent Active phase labor arrest Primary arrest of labor Secondary arrest of labor **Dystocia** Dystocic labor Labor dystocia Cervical dystocia Vaginal dystocia Functional dystocia Oxytocin-resistant dystocia Dynamic dystocia Mechanic dystocia True labor dystocia Dystocia with effective uterine activity Dystocia with ineffective uterine activity Primary dystocia Secondary dystocia	**Protraction** Protraction disorders Protracted labor Protracted active phase of labor Protracted first stage of labor Protracted second stage of labor Protracted descent Mildly protracted active phase Very protracted active phase **Progress** Failure to progress (often FTP) Poor (labor) progress Slow labor progress Nonprogression of labor (often NPOL) Nonprogressive labor **Delay** Delayed labor Delay in first stage of labor Delay in second stage of labor **Abnormal** Abnormal labor (progress) Abnormally prolonged labor Abnormally slow labor Abnormal dilation/dilatation **Other** Obstructed labor Cephalopelvic disproportion (CPD) Uterine inertia Failed labor Dysfunctional labor

### 2. Defining studies

We identified 43 studies that focused on defining a prolonged first and/or second stage of labor as primary outcome. ^14^^,^^17^^,^^31^^,^^35^^,^^39^, ^40^, ^41^, ^42^, ^43^, ^44^, ^45^, ^46^, ^47^, ^48^, ^49^, ^50^, ^51^, ^52^, ^53^, ^54^, ^55^, ^56^, ^57^, ^58^, ^59^, ^60^, ^61^, ^62^, ^63^, ^64^, ^65^, ^66^, ^67^, ^68^, ^69^, ^70^, ^71^, ^72^, ^73^, ^74^, ^75^, ^76^, ^77^ All of these studies, except four, originated from North America or Europe.^44^^,^^72^^,^^73^^,^^77^ The majority were observational studies (35, 81.4%). Table 2 (page 41) is an overview of all definitions provided by these “defining” studies.

**Table 2:** Forty-three studies defining prolonged labor

					First and second stage defined
Study Origin	Study design	Population	Wording	Definition prolonged first stage	Definition prolonged second stage
Bernitz 2019 NO^40^	RCT	7277 nulliparous	True labor dystocia	Two groups: (1) WHO group: partograph with 4 h action line crossed (2) Zhang group time intervals >p95 (calculated on basis of time intervals from 1 cm to the next). No significant difference in intrapartum CS	Two groups: (1) WHO group >1 h for head reaching pelvic floor or >1 h for expulsion (2 h in EA) (2) Zhang group 1 h 45 m descending (2 h30 EDA) or expulsion >1 h
Blankenship 2020 US^42^	Prospective cohort	6823 (reached second stage)	Prolonged first stage of labor	>90th (11.3 h), 95th (14.3 h) and 97th (17.0 h) centile of cohort. Benefit of expectantly managing prolonged first stage >90th centile must be weighed	3 h nulliparous with EA, 2 h nulliparous without EA, 2 h multiparous with EA, 1 h multiparous without EA
Hanzal 1993 AT^53^	Case-control study	107 nulli with CS for CPD	CPD	Diagnosis of CPD was established on vaginal examination by failing engagement of fetal head with severe molding while the cervix was fully dilated and/or marked prominence of the presenting part over the public symphysis	Diagnosis of CPD was established on vaginal examination by failing engagement of fetal head with severe molding while the cervix was fully dilated and/or marked prominence of the presenting part over the public symphysis
Hicklin 2019 US^55^	Other	Simulation models	Failure to progress	Allow to last longer than Friedman curves, possibly with different stopping rules at different dilatation moments. Labor duration >2 h (time limit for labor arrest based on the Friedman curve) should be allowed in each dilation state; furthermore, the allowed labor duration should be a function of dilation state	Not clear cut-off, analyzed per hour duration
Lu 2019 US^17^	Retrospective cohort	3079	Prolonged labor and dystocia	95th centile of cohort: 14.4 h	95th centile of cohort: 3.2 h in nulliparous with EA, 2.8 in nulliparous without EA, 1.6 h in multiparous with EA and 0.6 in multiparous without EA
Sizer 2000 GB^71^	Prospective cohort	1413	Dystocia	Progress of less than 1 cm of cervical dilatation per hour after 3 cm had been achieved (not an outcome of the study)	A second stage partograph was designed in this study, taking position and station into account, with a different partograph for nulli- and multiparous
Zhang 2010 US^14^	Retrospective cohort	62,415 with vaginal birth	Abnormal labor progression	95th centile of cohort. Take nonlinear relationship into account (4 h at early labor is normal but not after 6 cm). Duration depends on dilatation at admission, whereby curves were created for admission at 2,3,4, and 5 cm. The 95th centile of total labor duration were respectively 20.0, 17.4, 16.4 and 12.7 h	95th centile of cohort: 3.6 and 2.8 h nulliparous with and without EA, 2.0/1.3 for para 1, 1.6/1.1 for para 2+
Zhang 2002 US^76^	Retrospective cohort	1329 nulliparous with vaginal birth	Protraction and arrest disorders	From 4 to 10 cm, it takes approximately 5.5 h. 95th centiles of the time intervals suggest that labor lasting >2 h without perceivable change is not uncommon before 7 cm. The 5th centile indicates that in many patients the rate of change never exceeds 1 cm/h. 95th centile of total labor duration was not given, 90th centile was 13.7 h	At the second stage of labor, it may take up to 3 h to descend from station +1/3 to +2/3 and an additional 30 min to delivery

					Only first stage defined
Study	Study design	Population	Wording	Definition prolonged first stage	Definition prolonged second stage
Buchmann 2008 ZA^44^	Prospective cohort	504	CPD	CPD is a clinical diagnosis, in this study defined as CS for poor progress in labor. There is an association with sagittal suture overlap for diagnosis of CPD	
Govindappagari 2020 US^35^	Retrospective cohort	2559 nulliparous	Mildly and very protracted active phase	Mildly protracted: 1 cm or less in 4–6 h (≥6 cm) Very protracted 1 cm or less in 6 h	
Hamilton 2016 US^52^	Retrospective cohort	4703	Arrest of dilatation	Percentiles for dilatation and station	
Harper 2014 US^54^	Retrospective cohort	5030	Abnormal first stage	90th, 95th and 97th centiles. 90th: 10.33 h (multiparous spontaneous labor)—13.00 h (nulliparous induced), 95th: 12.55–15.50 h, 97th: 14.88–17.33 h	
Kawakita 2021 US^57^	Retrospective cohort	31,505 nulliparous	Arrest of dilation	Defined as ACOG guidelines. But at 6–7 cm allowed to wait ≥4 h since not associated with adverse neonatal outcomes	
Lavender 1998 GB^58^	RCT	928 nulliparous	Prolonged labor	WHO partograph (1 cm/h), prolonged labor is action line crossed (3 types, 2/3/4 h)	
Lavender 1999 US^59^	Prospective cohort	615 nulliparous	Prolonged labor	WHO partograph (1 cm/h), prolonged labor is action line crossed (3 types, 2/3/4 h). Women in 2 h arm more satisfied with labor experience	
Lavender 2005 GB^60^	Prospective cohort	403 multiparous	Dystocia	It is difficult to recommend universal definitions of failure to progress and we are unable to say when long labor should be acted upon. The proposed centile values may assist in the definition of dystocia. Slowest 5th centile=0.5 cm/h, 10th centile 0.7 cm/h	
Lavender 2006 GB^61^	RCT	2975 nulliparous	Prolonged labor	Crossing the action line of the WHO partograph, 2 vs 4 h	
Melman 2016 NL^63^	Other (Delphi)	2060 women 18 O&G	Nonprogression	2–4 h no progress after all measures taken (ROM, catheterization, pain relief, adequate contractions)	
Neal 2016 US^64^	Other	34 nulliparous	Labor dystocia	Based on the dystocia line of the Neal & Lowe partograph (stepwise with different cut-offs per cm dilatation)	
Ragusa 2021 IT^67^	Survey	289 midwives	Dystocia in labor	76.8% of midwives investigated agree there is currently no unambiguous definition of labor dystocia, 12.1% would define dystocia as a lack of cervical dilation for more than 2 h and 11.1% think that dystocia occurs when labor proceeds at less than 1.2 cm/h in nulliparous women	
Romijn 2016 NL^68^	Survey	147 healthcare workers	Prolonged first stage of labor	No clear definition but factors of influence: cervical dilatation, women's state of mind, intensity of contractions	
Rouse 1999 US^69^	Prospective cohort	542	Active phase labor arrest	1 cm or less of cervical progress in 2 h. Extend minimum period of oxytocin from 2 to 4 h	
Rouse 2001 US^70^	Prospective cohort	501	Active phase labor arrest	4 h (if >200 MVU) or 6 h (if <200 MVU) before CS for labor arrest. Majority of women (61%) who experience 2 h of labor arrest despite a sustained uterine contraction pattern of at least 200 Montevideo units will achieve vaginal delivery if oxytocin augmentation is continued. What is the definition?	
Souza 2018 NG, UG^72^	Prospective cohort	9995	Prolonged labor, obstructed labor	WHO partograph >1 cm/h alert line. Poor predictor of severe outcomes, advice to be revised	
Spörri 1997 CH^73^	Interventional study	41	Dystocia (CPD and failure to progress)	CPD was defined as arrest of labor for more than 4 h despite normal uterine contractions (5–7 per 15 min, duration 30–60 s). FTP similar but without adequate contractions. Progress of labor was considered inadequate as defined by cervical dilation of <2 cm over 2–3 h or lack of descent of the fetal head over 2–3 h	
White 2017 US^75^	Survey	664 healthcare workers	Dystocia, obstructed labor, active phase arrest	<0.5–2 cm/h (different responses)	

					Only second stage defined
Study	Study design	Population	Wording	Definition prolonged first stage	Definition prolonged second stage
Ausbeck 2020 US^39^	Retrospective cohort	218	Prolonged second stage of labor		4 h (with EA)/3 h (without EA) for nulliparous and 3 h (with EA)/2 h (without EA) for multiparous or 3 h/2 h/2 h/1 h. In this study, the latter was evaluated, with increased adverse maternal outcomes if expectant management was practiced beyond these timeframes
Bergsjø 1980 NO^31^	Prospective cohort	635 women in second stage	Prolonged second stage of labor		45 min nulliparous, 35 min multiparous
Blanc-Petitjean 2020 FR^41^	Retrospective cohort	177 with vaginal birth	Prolonged active second stage of labor		Extending >45 min is reasonable, for active second stage. Pushing started after maximum 3 h of full dilatation
Bleich 2012 US^43^	Retrospective cohort	21,991 nulliparous	Prolonged second stage of labor		>3 h in nulliparous women
Cheng 2004 US^45^	Retrospective cohort	15,759 nulliparous	Prolonged second stage of labor		>4 h (extremely prolonged second stage labor). In introduction of study also the ACOG guideline of 3/2/2/1 h mentioned
Cheng 2007 US^46^	Retrospective cohort	5158 multiparous	Prolonged second stage of labor		>3 h (multiparous). 2 h/1 h for multiparous mentioned
Cheng 2011 US^47^	Retrospective cohort	7558 nulli with vaginal birth	Prolonged second stage of labor		Multiparous >3 h leads to increased risks. Definition of 3 h/2 h/2 h/1 h mentioned
Finnegan 2019 IE^48^	Prospective cohort	2336 nulliparous	Prolonged second stage of labor		Limiting to 2 h in nulliparous patients: 1 h passive, 1 h active
Gimovsky 2016 US^49^	RCT	78 nulliparous	Prolonged second stage of labor		>2 h nulliparous without EA, >3 h with EA. Extended group >3 h/>4 h, no significant difference on outcomes, less cesarean sections
Gimovsky 2022 US^50^	Survey	34 nulliparous	Prolonged second stage of labor		>3 or 4 h, extension no impact on pelvic floor dysfunction
Grobman 2016 US^51^	Retrospective cohort	53,285	Arrest of second stage of labor		2 h/3 h, longer duration on individualized basis
Kadar 1986 GB^56^	Retrospective cohort	410 nulli with vaginal birth	Second stage duration		3 h, except for young women with GA < 40 w
Le Ray 2011 FR^62^	Prospective cohort	3330 nulli with vaginal birth	Prolonged active/passive second stage of labor		Active second stage usually maximum of 30 min, optimum needs to be found
O'Connell 2003 GB^65^	Case-control study	364 nulliparous	Prolonged second stage of labor		Long second stage >2 h, but compared up to 6 h
Pham 2022 FR^66^	Survey	8154	Prolonged second stage of labor		Without EA: 89.6 min for nulliparous (90th centile) and 30.6 min multiparous. With EA: exceeding 3 h in nulliparous and 2 h in multiparous (185.7 min and 120.1 min)
Stephansson 2016 SE^74^	Retrospective cohort	72,593 with vaginal birth	Prolonged second stage of labor		2–3 h nulliparous, 1–2 h multiparous
Zipori 2019 IL^77^	Retrospective cohort	19,831	Second stage arrest		Previous nulliparous 3 h (with EA)/2 h (without EA), multiparous 2 h/1 h (after 1 h passive), new 4 h/3 h and 3 h/2 h

Country codes following ISO 3166-1: AT = Austria, CH = Switzerland, FR = France, GB = United Kingdom, IE = Ireland, IL = Israel, IT = Italy, NG = Nigeria, NL = The Netherlands, SE = Sweden, UG = Uganda, US = United States of America.

ACOG = American College of Obstetricians and Gynecologists, cm = centimeters, CPD = cephalopelvic disproportion, CS = cesarean section, EA = epidural analgesia, GA = gestational age, h = hours, min = minutes, MVU = Montevideo Units, PPH = postpartum haemorrhage, RCT = Randomized controlled trial, ROM = rupture of membranes, w = weeks, WHO = World Health Organization.

### Prolonged first stage of labour

**Figure 3:**
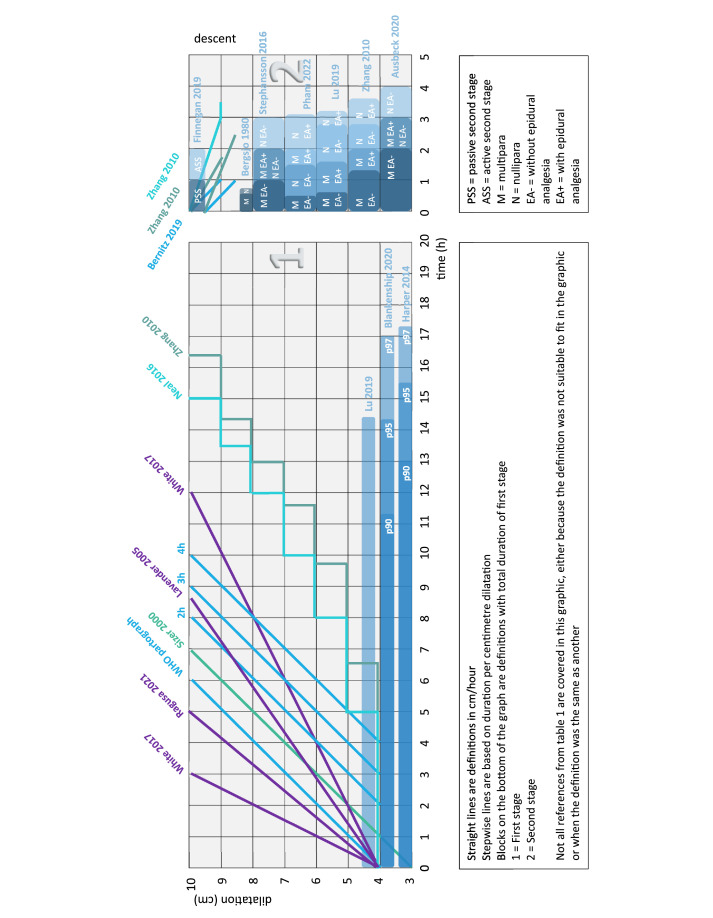
Graphic overview of definitions used in the studies depicted in Table 2.

In most studies, prolonged first stage of labor is defined based on a partograph, with time on the *X*-axis and progression of cervical dilatation on the *Y*-axis. Originally this had a one-centimeter-per-hour progress line (“alert line”) with a parallel action line 2 to 4 hours later.^40^^,^^58^^,^^61^ This partograph was assessed in five studies, whereby crossing the 4-hour action line was most commonly used to define prolonged labor.^61^ Some studies allow different slopes, either slower or faster than 1 cm/hour, while others allow shorter or longer periods of total time for active labor based on 90th, 95th or 97th percentiles of labor duration. Finally, especially more recent studies use adjusted time periods per centimeter of dilatation (stepwise approach).^14^^,^^64^ (see Figure 3).

Bakker. Scoping review on definition of prolonged labor. Am J Obstet Gynecol Glob Rep 2024.

### Prolonged second stage of labour

The definitions of prolonged second stage of labor are almost exclusively based on absolute times, originally allowing 2 hours of second stage for nulliparous and 1 hour for multiparous women.^48^^,^^65^^,^^74^ Most studies add an extra hour if epidural analgesia is used.^42^^,^^49^^,^^77^ More recent studies allow up to four or even 6 hours for the second stage and some distinguish between the descending phase (sometimes up to 3 hours) and the actively pushing second stage (also defined as passive vs active second stage).^49^, ^50^, ^51^ In some regions, time allowed for actively pushing is limited, ranging from 30 minutes to 1 hour.^31^^,^^41^^,^^48^ The partograph does not appear to play a major role in the second stage, although one study suggests a partograph specifically for the second stage, or including a specific part pertaining to the second stage.^71^

### 3. Other studies

We identified 189 studies with study objectives other than arriving at a definition of prolonged labor, but which specified a definition of prolonged labor or a synonym.^1^, ^2^, ^3^^,^^22^^,^^28^^,^^32^, ^33^, ^34^^,^^36^, ^37^, ^38^^,^^78^, ^79^, ^80^, ^81^, ^82^, ^83^, ^84^, ^85^, ^86^, ^87^, ^88^, ^89^, ^90^, ^91^, ^92^, ^93^, ^94^, ^95^, ^96^, ^97^, ^98^, ^99^, ^100^, ^101^, ^102^, ^103^, ^104^, ^105^, ^106^, ^107^, ^108^, ^109^, ^110^, ^111^, ^112^, ^113^, ^114^, ^115^, ^116^, ^117^, ^118^, ^119^, ^120^, ^121^, ^122^, ^123^, ^124^, ^125^, ^126^, ^127^, ^128^, ^129^, ^130^, ^131^, ^132^, ^133^, ^134^, ^135^, ^136^, ^137^, ^138^, ^139^, ^140^, ^141^, ^142^, ^143^, ^144^, ^145^, ^146^, ^147^, ^148^, ^149^, ^150^, ^151^, ^152^, ^153^, ^154^, ^155^, ^156^, ^157^, ^158^, ^159^, ^160^, ^161^, ^162^, ^163^, ^164^, ^165^, ^166^, ^167^, ^168^, ^169^, ^170^, ^171^, ^172^, ^173^, ^174^, ^175^, ^176^, ^177^, ^178^, ^179^, ^180^, ^181^, ^182^, ^183^, ^184^, ^185^, ^186^, ^187^, ^188^, ^189^, ^190^, ^191^, ^192^, ^193^, ^194^, ^195^, ^196^, ^197^, ^198^, ^199^, ^200^, ^201^, ^202^, ^203^, ^204^, ^205^, ^206^, ^207^, ^208^, ^209^, ^210^, ^211^, ^212^, ^213^, ^214^, ^215^, ^216^, ^217^, ^218^, ^219^, ^220^, ^221^, ^222^, ^223^, ^224^, ^225^, ^226^, ^227^, ^228^, ^229^, ^230^, ^231^, ^232^, ^233^, ^234^, ^235^, ^236^, ^237^, ^238^, ^239^, ^240^, ^241^, ^242^, ^243^, ^244^, ^245^, ^246^, ^247^, ^248^, ^249^, ^250^, ^251^, ^252^, ^253^, ^254^, ^255^ These definitions often correspond to one of the definitions given above. Out of the 189 studies, 154 reported a definition for prolonged first stage and 103 a definition for prolonged second stage (68 studies reported definitions for both).

### Prolonged first stage of labour

Table 3 provides an overview of all definitions used for prolonged first stage of labor, grouped into different categories. Most common are time-based definitions, whereby in older studies often an absolute maximum time was given for the total labor, sometimes counted from the time of admission to the labor ward, but usually from the start of established active labor.^22^^,^^94^^,^^100^^,^^127^^,^^130^, ^131^, ^132^^,^^155^^,^^158^^,^^166^^,^^170^^,^^187^^,^^188^^,^^194^^,^^199^^,^^209^^,^^234^^,^^236^^,^^237^^,^^239^^,^^243^ Some studies use separate definitions for nulliparous and multiparous women.^96^^,^^133^^,^^190^^,^^226^ In more recent studies, the definition of the American College of Obstetricians and Gynecologists (ACOG) and Society for Maternal-Fetal Medicine (SMFM) is often used to determine the time when a cesarean section for prolonged labor should be performed: at least six centimeters with ruptured membranes, 4 hours no progress with adequate uterine activity or 6 hours oxytocin administration with inadequate uterine activity and no cervical change. This definition does not distinguish between nulliparous and multiparous women. It is noted that this definition already includes the use of oxytocin in case of inadequate uterine contractions, and therefore as next step cesarean section is recommended.

**Table 3:** An overview of the definitions of prolonged first stage of labor

Category	Definition prolonged first stage of labor	Origin/reference
Time-based definitions: total labor duration	>8 h (multiparous women)^158^	Not specified
	>10 h^94^^,^^199^	Not specified
	>12 h^100^^,^^130^^,^^131^^,^^155^^,^^158^^,^^188^^,^^194^^,^^209^^,^^234^^,^^236^^,^^237^	WHO, NICE guideline
	>12 h with specifications: From time to admission^127^^,^^170^ With good contractions^187^	Not specified
	>14 h (multiparous women)^243^	Not specified
	>16 h^166^	Not specified
	>18 h^22^^,^^132^	Greenberg (2006), Allen (2009), Phillpott and Castle (1972)
	>20 h^239^^,^^243^	Not specified
	>24 h^79^^,^^90^^,^^93^^,^^173^^,^^174^^,^^184^^,^^220^^,^^227^^,^^244^	IMPAC guidelines
Time-based definitions: duration arrest	>1 h^163^	Not specified
	>2 h^34^^,^^36^^,^^104^^,^^105^^,^^107^^,^^117^^,^^123^^,^^125^^,^^141^^,^^160^^,^^169^^,^^183^^,^^185^^,^^197^^,^^203^^,^^210^, ^211^, ^212^^,^^214^^,^^215^^,^^226^^,^^247^	RTCOG, local guidelines
	>3 h^125^^,^^129^	Not specified
	>4 h^3^^,^^95^^,^^137^^,^^178^^,^^198^^,^^213^^,^^232^^,^^233^^,^^242^^,^^252^^,^^254^	Canada consensus conference, national guidelines, Obstetric care consensus, Zhang (2010)
	At least 6 cm with ruptured membranes, 4 h no progress with adequate uterine activity or 6 h oxytocin administration with inadequate uterine activity and no cervical change^83^^,^^91^^,^^104^^,^^112^^,^^113^^,^^124^^,^^149^^,^^182^^,^^221^^,^^229^^,^^248^^,^^249^	ACOG and SMFM
Progress-based: Dilatation per hour	<1.2 cm/h for 2 h^96^^,^^133^^,^^168^^,^^190^^,^^226^^,^^247^ Multiparous women <1.5 cm/h^96^^,^^133^^,^^169^^,^^190^^,^^226^	Friedman (1955), O'Driscoll (1973), WHO
	<1 cm/h^34^^,^^81^^,^^82^^,^^98^^,^^144^^,^^145^^,^^150^^,^^181^^,^^192^^,^^209^, ^210^, ^211^, ^212^^,^^223^^,^^227^	NICE guidelines, WHO
	<1 cm/2 h^207^	Not specified
	<2 cm/4 h^117^, ^118^, ^119^, ^120^^,^^126^^,^^143^^,^^153^^,^^181^^,^^193^^,^^200^^,^^204^^,^^233^	ACOG, Danish society, Fraser (1995), SOGC guidelines
Progress-based: With partograph	Crossing of action line, time not specified^140^^,^^145^^,^^191^	Not specified
	WHO partograph 2 h action line crossed ^101^^,^^151^^,^^152^^,^^156^^,^^217^^,^^230^^,^^246^	National guidelines, WHO, Philpott and Castle (1972)
	WHO partograph 4 h action line crossed^1^^,^^2^^,^^37^^,^^85^^,^^89^^,^^109^^,^^110^^,^^118^, ^119^, ^120^, ^121^^,^^144^^,^^166^^,^^206^^,^^223^^,^^231^^,^^238^	National guidelines, WHO, Philpott and Castle (1972)
	Neal & Lowe physiologic partograph^180^	Neal (2016)
	e-Partograph (digitally filled, unspecified)^146^	Sangvhi (2019)
	WHO partograph, unspecified. Alert line crossed^116^	WHO
	Partograph not specified^106^	Not specified
Clinically-based definitions	Hypocontractility^196^	Not specified
	Clinically diagnosed prolonged labor^122^^,^^245^	Not specified
	Clinically diagnosed CPD^88^^,^^102^^,^^108^^,^^157^^,^^192^^,^^228^^,^^253^	Not specified
	Grossly abnormal pelvis or obvious fetal hydrocephalus^1^	Not specified
	Clinically diagnosed obstructed labor^150^^,^^250^	Not specified
Outcome-based/database definitions	ICD-9 or -10 codes^33^^,^^80^^,^^99^^,^^138^^,^^142^^,^^189^^,^^241^^,^^255^	ICD
	CS with indication prolonged labor^81^^,^^139^^,^^208^^,^^240^	Not specified
	Use of oxytocin^88^	Not specified
	Patient reports^220^^,^^244^	Not specified
Other definitions	Based on average of cohort or population (75th^243^, 90th ^78^^,^^167^^,^^219^of 95th^109^^,^^110^^,^^205^ percentile)	Not specified
	No change in dilatation, time not specified^28^^,^^186^	Not specified
	Abnormal labor curve, type not specified^225^	Not specified
	Several diagnoses, no specific cut-off^38^^,^^97^	Not specified
	X-ray or pelvimetry diagnoses^216^	Not specified

ACOG = American College of Obstetricians and Gynecologists, AVB = assisted vaginal birth, cm = centimeter, CPD = cephalopelvic disproportion, CS = cesarean section, h = hours, ICD = International Classification of Diseases, IMPAC = Integrated Management of Pregnancy and Childbirth Care, NICE = National Institute for Health and Care Excellence, RTCOG = Royal Thai College of Obstetricians and Gynaecologists, SOGC = Society of Obstetricians and Gynaecologists of Canada, SMFM = Society for Maternal-Fetal Medicine, WHO = World Health Organization.

For studies into assessing quality of care or adherence to guidelines, labor curve-based definitions are often used, most often the WHO partograph with a 4-hour action line. In total, 72 out of 189 studies mentioned the partograph in the definition of prolonged labor. In some studies, specific additional criteria are required before performing a cesarean section, such as ruptured membranes, trial of oxytocin, or information on the efficacy of contractions. There is discrepancy between studies on next steps that are advised after establishing prolonged labor. If oxytocin augmentation is a criterion considered in defining prolonged labor, then cesarean section is generally recommended as the next step. Other studies, however, consider the establishment of prolonged labor as the moment to start oxytocin augmentation. Seventy studies state that the diagnosis of prolonged labor cannot be established without the presence of adequate uterine contractions and include this as part of the definition, where either classified in Montevideo units or based on frequency and duration of the contractions. Fifty-three studies mention the need for presence of ruptured membranes or amniotomy specifically before prolonged labor can be diagnosed.

Retrospective studies often make use of database-coded definitions, for example, the International Classification of Disease Ninth or Tenth revision (ICD-9 or -10), or indications filled in patient files.^138^^,^^142^^,^^189^^,^^241^ Thirteen studies used clinically-based definitions, which sometimes did not entail more than “defined as per decision of the clinician,” but in some cases are based on clinical features such as severe molding or failed engagement. Three studies made use of patient reports for the definition of prolonged labor.^79^^,^^220^^,^^244^ Instead of labor dynamics, some, mainly older studies focus solely on CPD and obstructed labor, diagnosed upon clinical/pelvic examination. The reliability of this examination can be questioned.^97^

### Prolonged second stage of labour

Table 4 provides an overview of all definitions for prolonged second stage of labor. Most definitions for prolonged second stage of labor are time-based with different limits for nulliparous and multiparous women. In 60 studies the total duration of the second stage was given a time limit, varying from 30 minutes to the most recent ACOG definition of at least 4 hours of dilatation for nulliparous women with an epidural, 3 hours for nulliparous women without epidural, 3 hours for multiparous women with epidural and 2 hours for multiparous women without epidural.^116^^,^^256^ Other studies did not apply a time limit to the total duration of the second stage, but to the period of time that no progress (descent of the presenting part) was observed. Ramm et al.^202^ argues that there should be no limit to the total duration of the second stage as long as progress in descent is documented. Some studies are focused on the role of ultrasound in the assessment of progress or diagnosis of prolonged second stage of labor.^118^, ^119^, ^120^^,^^148^ This could be assessment of the position of the fetal head or the relation to the pelvis. As with prolonged first stage, there are also outcome-based definitions used for prolonged second stage, in some studies identified as (failed) instrumental birth.

**Table 4:** An overview of the definitions of prolonged second stage of labor

Category	Nulliparous	Multiparous	Origin
Total duration	>2 h^3^^,^^81^^,^^129^^,^^136^^,^^147^^,^^168^^,^^199^^,^^217^^,^^227^		O'Driscoll (1973)
	>3 h^188^^,^^232^^,^^252^		French guidelines, LeRay
	>1 h^109^^,^^110^	>30 min^238^	Philpott and Castle (1972)
	>2 h^2^^,^^96^^,^^114^^,^^156^^,^^162^^,^^177^^,^^179^	>1 h^2^^,^^96^^,^^114^^,^^156^^,^^162^^,^^179^	WHO, local guidelines
	>3 h^92^^,^^107^^,^^112^^,^^113^^,^^132^^,^^154^^,^^178^^,^^201^^,^^215^^,^^243^	>2 h^107^^,^^112^^,^^113^^,^^132^^,^^154^^,^^243^	ACOG & SMFM
	>4 h^222^, very protracted >7 h (95th centile cohort)		Spong (2012)
	>2 h without EA >3 h with EA^84^^,^^86^^,^^87^^,^^133^, ^134^, ^135^^,^^159^^,^^161^^,^^164^^,^^165^^,^^167^^,^^171^^,^^195^^,^^210^, ^211^, ^212^^,^^218^^,^^224^^,^^228^^,^^235^^,^^242^^,^^251^	>1 h without EA >2 h with EA^87^^,^^133^, ^134^, ^135^^,^^159^^,^^161^^,^^165^^,^^167^^,^^171^^,^^195^^,^^210^, ^211^, ^212^^,^^218^^,^^228^^,^^235^^,^^251^	ACOG & SMFM
	>3 h without EA >4 h with EA^128^^,^^137^^,^^221^^,^^229^	>2 h without EA >3 h with EA^137^^,^^229^	ACOG & SMFM, Spong (2012)
	1 h 45 with EA 2 h 30 m without EA^109^^,^^110^		Zhang (2010)
Expulsion duration (or active second stage)	30 min^116^		Dujardin (1995)
	45 min^148^		Not specified
	1 h^126^^,^^153^^,^^183^^,^^198^^,^^206^^,^^214^^,^^236^		Local guidelines, NICE guidelines
	2 h^111^		ACOG
	3 h^149^		ACOG
	1 h^101^^,^^103^	30 m^101^^,^^103^	Local guidelines
	2 h^172^	1 h^172^	NICE, Caughey (2014)
	3 h^248^	2 h^248^	ACOG & SMFM, Zhang (2010)
	>3 h without EA >4 h with EA^249^	>2 h without EA >3 h with EA^249^	ACOG & SMFM
Arrest of descent	30 m^209^		Not specified
	1 h^36^^,^^175^^,^^226^		Local guidelines
	2 h^1^^,^^82^	1 h^1^	WHO, NICE guidelines
	3 h^95^^,^^126^^,^^153^	2 h^126^^,^^153^	Not specified
	>2 h without EA >3 h with EA^115^^,^^123^	>1 h without EA >2 h with EA^115^	ACOG & SMFM
	>3 h without EA >4 h with EA^83^^,^^91^	>2 h without EA >3 h with EA^83^^,^^91^	ACOG & SMFM
	Descent <1 cm/h^81^^,^^156^		Not specified
	Failure to descend^98^^,^^230^^,^^254^		O'Driscoll (1973)
Clinically based definitions	Checkboxes^204^		Fraser (1995)
	No limit as long as progress is observed^202^		Not specified
Outcome-based definitions	ICD codes^33^^,^^142^^,^^241^^,^^255^		ICD
	Instrumental birth performed^245^		Not specified
	Instrumental birth failed^108^^,^^140^^,^^198^		Not specified
Other definitions	2 h passive^32^^,^^94^		Not specified
	Crossing of action line on partograph^151^^,^^152^		WHO partograph

ACOG = American College of Obstetricians and Gynecologists, cm = centimeter, EA = epidural analgesia, h = hours, ICD = International Classification of Diseases, m = minutes, NICE = National Institute for Health and Care Excellence, SMFM = Society for Maternal-Fetal Medicine, WHO = World Health Organization.

## Discussion

### Principal findings

This study identified 53 synonyms for prolonged labor in the scientific literature, and a multitude of nonaligned definitions of prolonged labor were identified. As expected, there is extreme heterogeneity in these definitions. To increase knowledge on establishing and managing prolonged labor, common language, and uniform definitions are needed, both in clinical practice guidelines and in clinical research.

In the most recent literature, the use of definitions by ACOG and SMFM appears to be most common, taking the predominance of northern American studies into account.^256^ These definitions, and the studies they are based on, also form the basis for other national guidelines.^225^ These and other recent definitions seem to allow for more time for both first and second stage of labor, mostly based on the research performed by Zhang et al. Labor progression curves from these studies provide a stepwise approach to labor progress and allow for more time in labor than the original Friedman curves, which most of the older included studies were based on.^10^^,^^14^^,^^76^ Several comparisons have been conducted between the WHO partograph and the contemporary labor curves, whereby Bernitz et al. showed no difference in the intrapartum cesarean section rate and in proportion of oxytocin use.^40^ This study defined prolonged labor as crossing of the action line in the WHO partograph or at crossing of Zhang's curve, followed by an intervention based on local guidelines, whereby oxytocin was started if contractions were considered ineffective.

Difficulties arise when some studies advise the start of oxytocin when the diagnosis of prolonged labor is made, whilst others already incorporated this aspect into the diagnosis and recommend cesarean section as a next step in the clinical management. Many studies agree that particular standards must be met before oxytocin is applied or cesarean section performed. The presence of ruptured membranes is sometimes stated as requirement for the establishment of active labor and therefore specifically included in some definitions of prolonged labor. In case of inadequate contractions, a trial of oxytocin augmentation is also considered necessary before labor progress is labeled prolonged, and an essential part of some of the identified definitions. Considering this, a mere comparison of labor curves is insufficient to create uniform advise on labor management, whereby consensus is needed as to which intervention should be performed at which moment. However, a generalizable labor pattern might not exist and many factors are of influence, such as parity (with different definitions of prolonged second stage for nulli- and multiparity), obstetric or medical history, onset of labor process, measurement of contractions, and fetal condition. Furthermore, access to healthcare and risk of cesarean section vary between settings, which could impact decisions on clinical management and decision on cesarean section. As long as a good fetal and maternal condition are ensured, however, room for expectant management can be allowed, with possible individualization.^14^^,^^16^^,^^20^^,^^40^

### Comparison with existing literature

Two previous systematic reviews structured evidence on prolonged labor, whereby definitions of onset of active labor and duration of first and second stage were compared.^24^^,^^257^ Neal et al.^24^ concluded the lack of a universal clear definitions for prolonged labor, based on a review of clinical practice guidelines. The review by He et al. was predominantly focused on labor progression curves but also included definitions of prolonged labor. He et al.^257^ concluded that a change of thinking regarding labor progression was witnessed in the last 20 years, but that a debate on the discrepancy between the amount of knowledge gained and the limited change in clinical practice remains. Our review is different from these earlier reviews since we provide a structured overview and categorization of all definitions used in the scientific literature. Our findings indicate a vicious circle of definitions: definitions of prolonged labor applied in clinical research are often directly adopted from clinical practice guidelines, though the clinical practice guidelines are often poorly supported research evidence. Many clinical practice guidelines are developed by consensus among a limited group of experts not always in the clinical frontline, and disseminated without pilot testing or postimplementation testing of effects and side-effects. Our findings may thereby be an actionable first step in structuring the evidence and opening a path toward uniformity. Moreover, we call for critical and cautious application of prolonged labor definitions that are truly based on the latest decades’ evidence from labor progression studies and basic science, in both clinical practice guidelines and scientific research.^15^^,^^20^^,^^258^^,^^259^ The current over-medicalization related to labor progression causes excessive harm and evidence-based implementation research is crucial.^260^ For instance, the ICD-11 guidelines from 2023 still define prolonged labor as progress less than one centimeter per hour for a minimum of 4 hours, which is far from current evidence and, if followed, would mean that half of all primigravidae were diagnosed with prolonged labor.^16^

### Strengths and limitations

A strength of this study is the very inclusive search strategy, aiming to enclose as many synonyms for prolonged labor as possible. Due to the systematic search and assessment of papers, we established a large database of definitions, spread across all continents. Nonetheless, there might be even other less common synonyms used for prolonged labor, which did not come out of our search. We acknowledge that important studies from Francophone countries or Latin America might have been missed due to the exclusive inclusion of English written literature and guidelines.

It could be argued that in this review we pooled too many different definitions related to prolonged labor and considered these as synonyms. While some studies made clear distinctions between certain terms, for example, between delay and arrest, or between various forms of dystocia, other studies use various terms interchangeably.^37^^,^^38^ This was also observed with the terms CPD and obstructed labor, which are generally considered as respectively a cause and end result of prolonged labor but are also sometimes used as synonyms for prolonged labor, which leads to considerable unclarity. We chose to include these terms in the review in order to be as comprehensive as possible, although these terms are not synonyms of prolonged labor. We strongly recommend that all clinical guidance clearly expresses that CPD is not a synonym of prolonged labor and that CPD, in fact, can only be identified by undergoing a trial of labor and only if other causes of prolonged labor (e.g., inaccurate contractions) are excluded. Moreover, we recommend that obstructed labor requires a clear and unequivocal definition to be identified as a specific end-stage of neglected prolonged labor with particularly severe consequences. Only then could the incidence, clinical course, and appropriate management of obstructed labor be studied robustly.

### Research implications

We believe that a first step could be to analyze the effects of applying various definitions on labor outcomes in various settings, which has already been done by a few researchers.^40^^,^^61^ Furthermore, we should aim for more understanding of the pathophysiology of prolonged labor and use this to explore possibilities for individual care. Notably, when creating the much-needed global definition of prolonged labor that is evidence-based and clinically useful, healthcare workers should be invited to influence this process and advise on which diagnostic tool to use in the establishment of the diagnosis. This should be aligned with clear and concise labor management advice, whereby the indication and moment of amniotomy, oxytocin, and proceeding to cesarean section or assisted vaginal birth should be clearly specified. There is a role for global organizations such as WHO and the International Federation of Gynaecology and Obstetrics to structure national and international guidelines. Standardization and consensus are beneficial for researchers, clinicians, and all women with prolonged labor, to ensure optimal treatment when necessary and prevent both over- and underuse of interventions, although at the same time, it is crucial to adapt such guidance to the local context.

## Conclusion

To date, a tremendous number of synonyms and definitions of prolonged first and second stage of labor are being used. Current definitions can be categorized into time-based, progress-based, clinically-based and outcome-based. Our findings indicate a vicious circle of definitions: definitions of prolonged labor applied in clinical research are often directly adopted from clinical practice guidelines, though the clinical practice guidelines are often poorly supported by research evidence. This lack of consistent use of terminology and definition hampers research into clinical decision-marking in prolonged labor. Clinicians and midwives are in need of uniform, evidence-based definitions accompanied by evidence-based and clear clinical guidance and we believe our results are a call for action to apply the available evidence on labor progression in clear, unequivocal clinical guidance that may be adapted to the local context.

## CRediT authorship contribution statement

**Wouter Bakker:** Writing – review & editing, Writing – original draft, Visualization, Validation, Supervision, Resources, Project administration, Methodology, Investigation, Formal analysis, Data curation, Conceptualization. **Evelien M. Sandberg:** Writing – review & editing, Methodology, Investigation, Formal analysis, Data curation, Conceptualization. **Sharon Keetels:** Writing – review & editing, Investigation, Formal analysis, Data curation. **Jan W. Schoones:** Writing – review & editing, Software, Resources, Investigation, Data curation. **Monica Lauridsen Kujabi:** Writing – review & editing, Visualization, Validation, Methodology. **Nanna Maaløe:** Writing – review & editing, Visualization, Validation, Supervision, Methodology. **Salome Maswime:** Writing – review & editing, Validation, Supervision. **Thomas van den Akker:** Writing – review & editing, Visualization, Validation, Supervision, Methodology, Investigation, Data curation, Conceptualization.

## Conflicts of Interest

The authors declare no conflict of interest, other than that some have contributed to included studies.^1^^,^^2^^,^^88^ SM is funded through a mid-career Scientist Award by the South African Medical Research Council.

## Appendix Supplementary materials

Appendix S1. Search strategy (separate file). Image, application 1

Appendix S2. PRISMA checklist (separate file). Image, application 2

## Footnotes

Supplementary material associated with this article can be found in the online version at doi:10.1016/j.xagr.2024.100360.
