# Supplementary material for: Inconsistent definitions of prolonged labor in international literature: a scoping review
Source: AJOG Glob Rep. 2024 Jun 5;4(3):100360. doi: 10.1016/j.xagr.2024.100360 (PMC11261896; doi:10.1016/j.xagr.2024.100360)
Supplement: Supplementary file 1 — Appendix S1. Search strategy (separate file). [file mmc1.docx]

**Appendix S1: Search strategy**

(("obstructed labour"[tw] OR "obstructed labours"[tw] OR "obstructed labor"[tw] OR "obstructed labors"[tw] OR "obstructed childbirth"[tw] OR "obstructed birth"[tw] OR "Cephalopelvic Disproportion"[Mesh] OR "Cephalopelvic Disproportion"[tw] OR "Cephalopelvic Disproportions"[tw] OR "prolonged first stage of labor"[tw] OR "prolonged first stage of labour"[tw] OR "prolonged first stage labor"[tw] OR "prolonged first stage labour"[tw] OR "prolonged second stage labor"[tw] OR "prolonged second stage labour"[tw] OR "prolonged second stage of labor"[tw] OR "prolonged second stage of labour"[tw] OR ("prolonged"[tw] AND ("1st"[tw] OR "2nd"[tw] OR "first"[tw] OR "second"[tw]) AND ("stage of labour"[tw] OR "stage of labours"[tw] OR "stage of labor"[tw] OR "stage of labors"[tw] OR "stage of labour"[tw] OR "stages of labours"[tw] OR "stages of labor"[tw] OR "stages of labors"[tw] OR "labor stage"[tw] OR "labour stage"[tw] OR "labor stages"[tw] OR "labour stages"[tw])) OR "cervical dystocia"[tw] OR "cervical dystocias"[tw] OR "cervix dystocia"[tw] OR "big fundus"[tw] OR "prolonged labour"[tw] OR "prolonged labor"[tw] OR ("delay*"[tw] AND ("1st phase"[tw] OR "1st stage"[tw] OR "2nd phase"[tw] OR "2nd stage"[tw] OR "first phase"[tw] OR "first stage"[tw] OR "second phase"[tw] OR "second stage"[tw]) AND ("Labor, Obstetric"[Mesh] OR "labor"[tw] OR "labors"[tw] OR "labour"[tw] OR "labours"[tw])) OR "arrest of labor"[tw] OR "arrest of labour"[tw] OR "labor arrest"[tw] OR "labour arrest"[tw] OR (("first-stage arrest"[tw] OR "second-stage arrest"[tw]) AND ("Labor, Obstetric"[Mesh] OR "labor"[tw] OR "labors"[tw] OR "labour"[tw] OR "labours"[tw])) OR ("Delivery, Obstetric"[majr] AND ("Pregnancy Complications"[majr:noexp] OR "Obstetric Labor Complications"[Majr:noexp] OR "Quality of Health Care"[majr]))) AND ("definition"[tw] OR "definitions"[tw] OR "defined"[tw] OR "define"[tw] OR "defining"[tw] OR "Terminology as Topic"[Mesh] OR "terminology"[tw] OR terminolog*[tw] OR "etymology"[tw] OR etymolog*[tw] OR "nomenclature"[tw] OR nomenclat*[tw] OR "Classification"[Mesh] OR "classification"[Subheading] OR "classification"[tw] OR classificat*[tw] OR classify*[tw] OR "systematics"[tw] OR taxonom*[tw] OR "Delivery, Obstetric/standards"[majr] OR (("Delivery, Obstetric/statistics and numerical data"[majr:noexp] OR "Labor, Obstetric/statistics and numerical data"[majr:noexp] OR "Cesarean Section/statistics and numerical data"[Majr]) AND "statistics and numerical data"[Subheading:NoExp]) OR ("criteria"[ti] AND audit*[ti]))) AND English[la]
